# Undernutrition: a major but potentially preventable cause of poor outcomes in children living with sickle cell disease in Africa

**DOI:** 10.1186/s12916-020-01892-4

**Published:** 2021-01-15

**Authors:** Thomas N. Williams

**Affiliations:** 1 Department of Epidemiology and Demography, KEMRI-Wellcome Trust Research Programme, PO Box 230, Kilifi, 80108 Kenya; 2 Faculty of Medicine, Imperial College, St Mary’s Hospital, London, W21NY UK

**Keywords:** Sickle cell disease, Malnutrition, Nutrition, Africa

## Background

Sickle cell disease (SCD) is a common but neglected condition in sub-Saharan Africa. Although up to 230,000 babies are born with SCD in the region every year [1], most countries lack comprehensive programmes for its early detection, which could direct these children towards the care they really need. This means that, despite the fact that most deaths could be avoided through the provision of just a few simple and affordable interventions [2, 3], historically, as many as 50–90% of children born in Africa with SCD have been dying before they have reached their 5th birthday [4]. More recently, a number of welcome initiatives have raised the profile of SCD in Africa and, as a result, an increasing number of children are now surviving and receiving treatment. In the meantime, through lack of research, significant gaps remain in our knowledge of the clinical epidemiology of SCD in Africa, rendering studies like that conducted by Islam and colleagues [5] particularly important.

## Nutritional status and sickle cell disease

For the first time in Africa, real-time SCD testing of children between six and 59 months of age (through use of the SickleSCAN™ rapid test) was included in the latest Nigerian Demographic Health Survey [6]. This gave Islam and colleagues the opportunity to analyse data relating to variables included in the survey with respect to SCD status. They focused their analysis on nutrition, finding that among the sub-group of children with SCD, the overall prevalence levels for stunting (a low height-for-age *z*-score), wasting (a low weight-for-height *z*-score), and underweight (a low weight-for-height *z*-score) were 55.4% (54.5–56.4%), 9.1% (8.6–9.7%), and 38.9% (38.0–39.8%), respectively [5]. Importantly, they also found that stunting was 2.39 times more common (adjusted odds ratio (aOR) 2.39; 95% CI 1.26–4.54) while underweight was 2.64 times more common (aOR 2.64; 95% CI 1.25–5.98) in children with SCD than in those without [5].

Although undernutrition has long been recognised as an important complication of SCD [7], this study has value as it is the first to quantify the problem in a nationally representative population sample, within a major African country. Moreover, because SCD was diagnosed through a survey, it is assumed that most children did not know their status at the time of testing, therefore making the results representative of the natural history of SCD in the absence of active patient management. The study shows that children with SCD are significantly disadvantaged from a nutritional perspective and suggests that implementing specific interventions aimed at optimising their nutrition could make a positive contribution to their general health.

While the aetiology of undernutrition in children with SCD is not completely understood (reviewed in reference [7]), known causes include increased energy requirements due to an elevated basal metabolic rate, altered metabolic pathways, the increased degradation and loss of nutrients, and reduced dietary intake, potentially from the anorexic effects of co-morbidities. In addition, as for all organ systems, the gut can be damaged in children with sickle cell disease through vasculopathic processes that include recurrent hypoxia-reperfusion injury induced by vaso-occlusive crises (VOC), which could also lead to reduced nutrient absorptive capacity. Recent studies suggest that VOCs initiate a cascade of interconnected changes in the gut that include enterocyte injury and apoptosis, local inflammation and immune activation, disruption of tight junction integrity, and an altered population and function of gut microbiota [8, 9]. On the background of this pathophysiological complexity, the analysis by Islam, which reported that the association of SCD with anthropometric indices was significantly and strongly mediated by haemoglobin levels, should be interpreted with some caution [5]. While certainly true from a purely statistical perspective, given the nature of this cohort study, the authors themselves indicate that they only had access to data on a very limited number of variables that could potentially be associated with nutritional status, making it impossible for them to evaluate the importance of haemoglobin levels in comparison to the many other potential mediators. Moreover, the cross-sectional design of their study did not allow them to draw conclusions about causality.

## Conclusions

This study provides important evidence about the frequency of undernutrition in children with SCD in Nigeria. However, it is not possible to conclude from this study that this issue could be mitigated by the optimization of their haemoglobin levels, without also addressing other potential aetiological causes. Further research into such causes, their relative mutual importance, and the development of targeted helpful interventions, should be a priority area for research going forwards.

## Data Availability

Not applicable
